# Supplementary material for: Emulating Hyperspectral and Narrow-Band Imaging for Deep-Learning-Driven Gastrointestinal Disorder Detection in Wireless Capsule Endoscopy
Source: Bioengineering (Basel). 2025 Sep 4;12(9):953. doi: 10.3390/bioengineering12090953 (PMC12467974; doi:10.3390/bioengineering12090953)
Supplement: Supplementary file 1 [file bioengineering-12-00953-s001.zip › bioengineering-3775991-supplementary.pdf]

bioengineering

MDPI

Supplementary

Emulating Hyperspectral and Narrow-Band Imaging for Deep Learning–Driven Gastrointestinal Disorder Detection in Wireless Capsule Endoscopy

Chu-Kuang Chou [1,2], Kun-Hua Lee [3,4], Riya Karmakar [4], Arvind Mukundan [4,5], Pratham Chandraskhar Gade [6], Devansh Gupta [7], Chang-Chao Su [1], Tsung-Hsien Chen [8], Chou-Yuan Ko [9,*], and Hsiang-Chen Wang [4,10,*]

1 Division of Gastroenterology and Hepatology, Department of Internal Medicine, Ditmanson Medical Foundation Chia-Yi Christian Hospital, Chia-Yi 60002, Taiwan; vacinu@gmail.com (C.-K.C.); 06155@cych.org.tw (C.-C.S.)
2 Obesity Center, Ditmanson Medical Foundation Chia-Yi Christian Hospital, Chia-Yi 60002, Taiwan.
3 Department of Trauma, Changhua Christian Hospital, Changhua; No.135, Nanxiao St., Changhua City, Changhua County, 50006, Taiwan, 88847@cch.org.tw (K.-H.L.)
4 Department of Mechanical Engineering, National Chung Cheng University, 168, University Rd., Min Hsiung, Chia Yi 62102, Taiwan; karmakarriya345@gmail.com (R.K.); arvindmukund96@gmail.com (A.M.)
5 Department of Biomedical Imaging, Chennai Institute of Technology, Sarathy Nagar, Chennai 600069, Tamil Nadu, India
6 Information Technology Department, Sanjivani College of Engineering, Kopargaon, India; pratham-gade2324_it@sanjivanicoe.org.in (P.C.G)
7 Computer Science and Engineering Department, Thapar Institute of Engineering & Technology, Patiala 147001, Punjab, India; dgupta1_be21@thapar.edu (D.G.)
8 Department of Internal Medicine, Ditmanson Medical Foundation Chia-Yi Christian Hospital, Chiayi 60002, Taiwan; cych13794@gmail.com (T.-H.C.)
9 Department of Gastroenterology, Kaohsiung Armed Forces General Hospital, 2, Zhongzheng 1st.Rd., Lingya District, Kaohsiung City 80284, Taiwan; gastroenterokjy@gmail.com (C.-Y.K.)
10 Department of Technology Development, Hitspectra Intelligent Technology Co., Ltd., Kaohsiung 80661, Taiwan
* Correspondence: gastroenterokjy@gmail.com (C.-Y.K.); hcwang@ccu.edu.tw (H.-C.W.)

Academic Editor: Firstname Lastname

Received: date
Revised: date
Accepted: date
Published: date

Citation: To be added by editorial staff during production.

Copyright: © 2025 by the authors. Submitted for possible open access publication under the terms and conditions of the Creative Commons Attribution (CC BY) license (https://creativecommons.org/licenses/by/4.0/).

Abstract: Diagnosing gastrointestinal disorders (GID) remains a significant challenge, particularly when relying on wireless capsule endoscopy (WCE), which lacks advanced imaging enhancements like Narrow Band Imaging (NBI). To address this, we propose a novel framework Spectrum-Aided Vision Enhancer (SAVE) especially designed to transform standard white light (WLI) endoscopic images into spectrally enriched representations that emulate both hyperspectral imaging (HSI) and NBI formats. By leveraging colour calibration through the Macbeth Colour Checker, gamma correction, CIE 1931 XYZ transformation, and principal component analysis (PCA), SAVE reconstructs detailed spectral information from conventional RGB inputs. Performance was evaluated using the Kvasir-v2 dataset, which includes 6,490 annotated images spanning eight GI-related categories. Deep learning models like ResNet50, MobileNetV2, MobileNetV3 and Alex Net were trained on both original WLI and SAVE-enhanced images. Among these, MobileNetV2 achieved an F1-score of 96% for polyp classification using SAVE and Alex Net saw a notable increase in average accuracy to 84% when applied to enhanced images. Image quality assessment showed high structural similarity (SSIM scores of 93.99% for Olympus endoscopy and 90.68% for WCE), confirming the fidelity of the spectral transformations. Overall, the SAVE framework offers a practical, software-based enhancement strategy that significantly improves diagnostic accuracy in GI imaging, with strong implications for low-cost, non-invasive diagnostics using capsule endoscopy systems.

Keywords: Spectrum aided visual enhancer; Wireless capsule endoscopy; Gastrointestinal diseases; Hyperspectral imaging; White light imaging; Narrow band imaging; Polyps; Oesophagitis; ulcerative colitis

S.1 Deep Learning Modules

S1.1 MobileNetV2

MobileNetV2 is a lightweight deep convolutional neural network architecture introduced by Google, designed for efficient performance on mobile and edge devices. It builds upon the original MobileNet by introducing:

Inverted Residuals: Rather than using traditional residual connections, MobileNetV2 uses thin bottleneck layers followed by depthwise convolutions and expands the number of channels at the output.

Linear Bottlenecks: Non-linearities are removed from the bottleneck layer to preserve information.

Depthwise Separable Convolutions: These drastically reduce the number of parameters and computations by splitting convolution into two parts—depthwise and pointwise.

These design choices make MobileNetV2 highly suitable for real-time inference on mobile platforms, making it ideal for low-latency stereo vision applications.

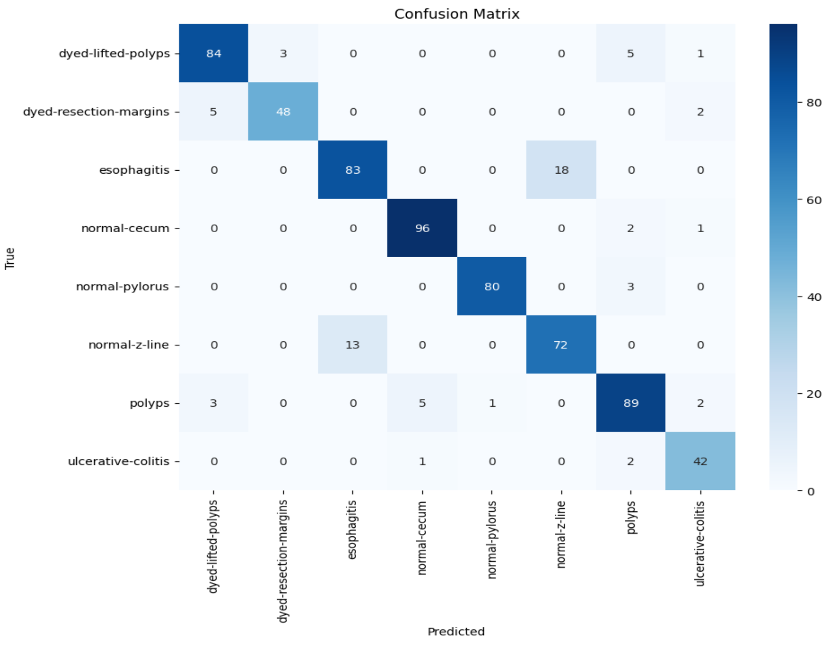

Figure S1. Confusion Matrix of MobileNetV2 for WLI image dataset

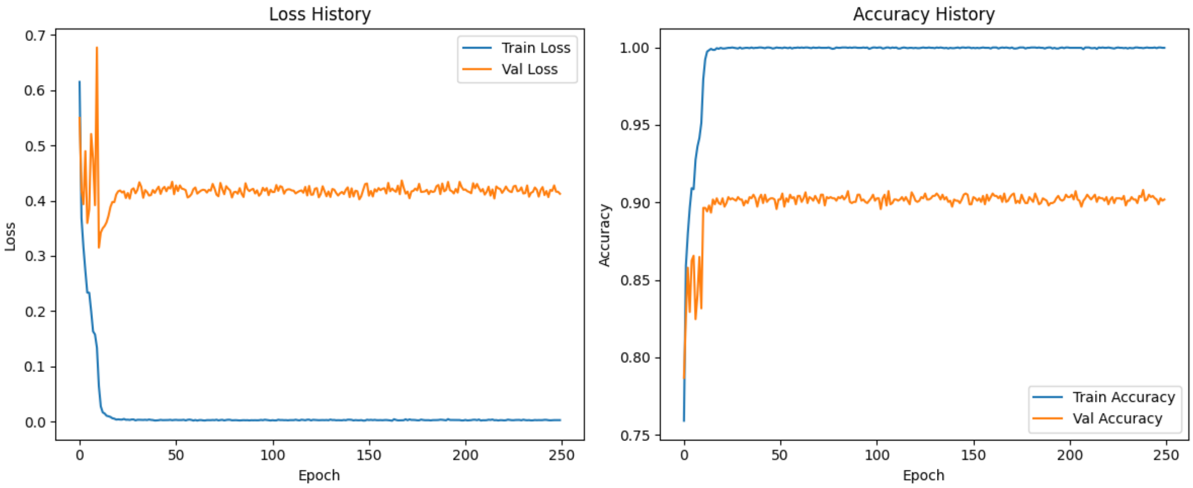

Figure S2. Loss and Accuracy History of MobileNetV2 for WLI image dataset

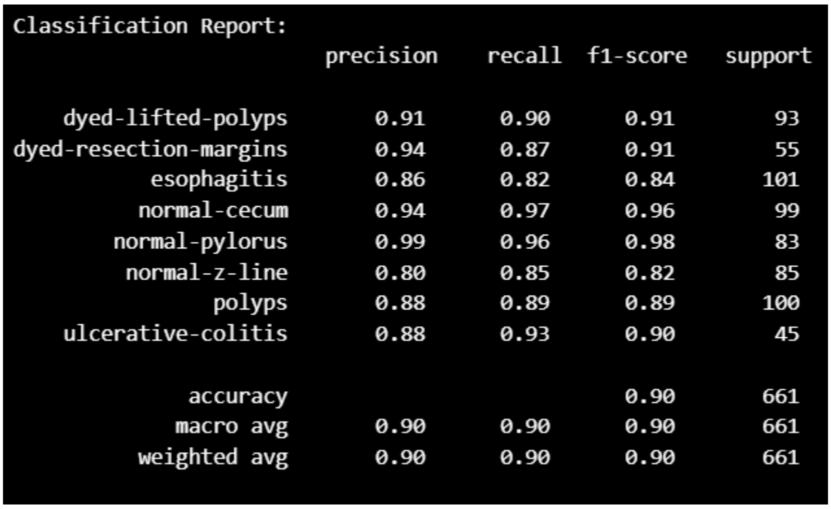

Classification Report:

	precision	recall	f1-score	support
dyed-lifted-polyps	0.91	0.90	0.91	93
dyed-resection-margins	0.94	0.87	0.91	55
esophagitis	0.86	0.82	0.84	101
normal-cecum	0.94	0.97	0.96	99
normal-pylorus	0.99	0.96	0.98	83
normal-z-line	0.80	0.85	0.82	85
polyps	0.88	0.89	0.89	100
ulcerative-colitis	0.88	0.93	0.90	45
accuracy			0.90	661
macro avg	0.90	0.90	0.90	661
weighted avg	0.90	0.90	0.90	661

Figure S3. Classification Report of MobileNetV2 for WLI image dataset

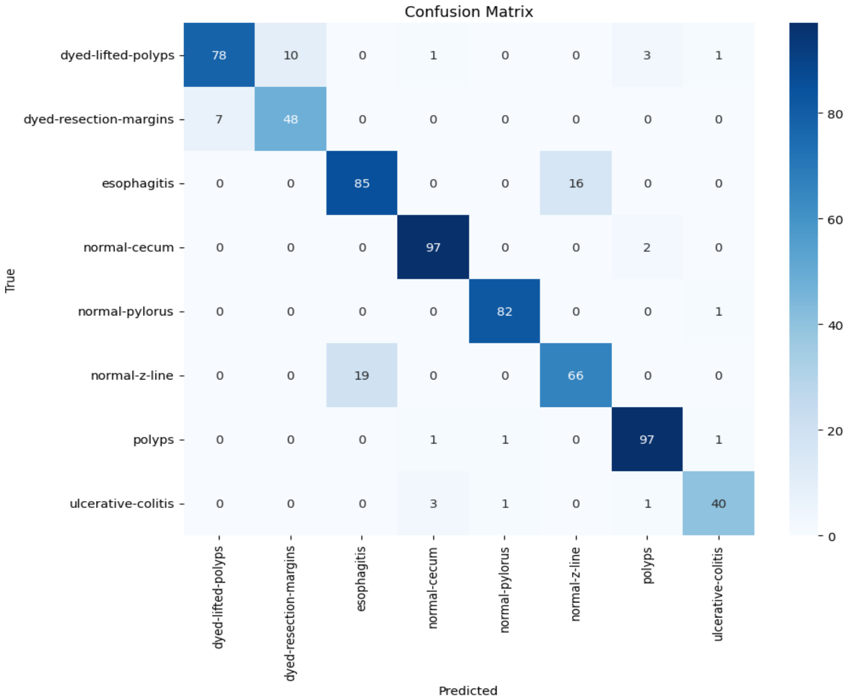

Figure S4. Confusion matrix of MobileNetV2 for SAVE image dataset

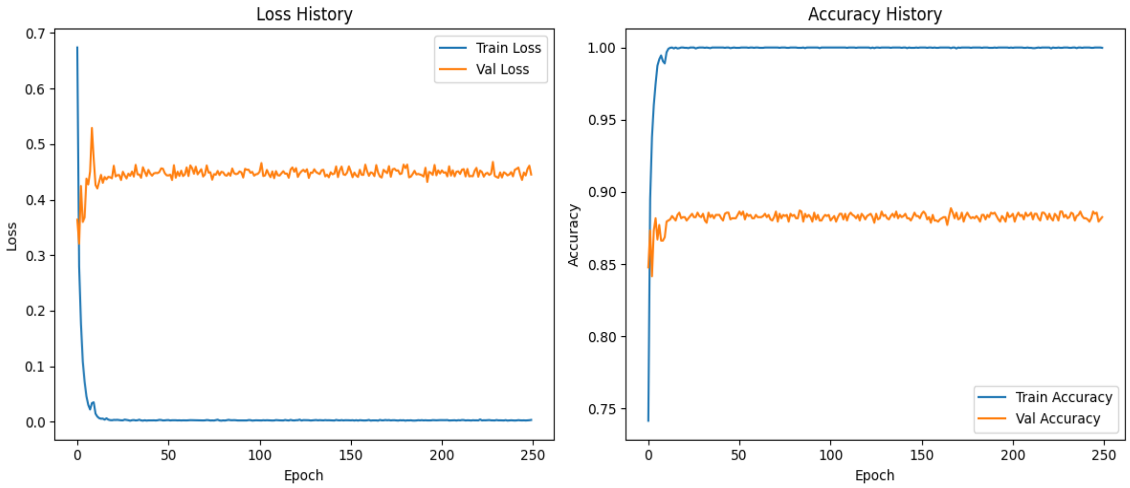

Figure S5. Loss and Accuracy History of MobileNetV2 for SAVE image dataset

Classification Report:

	precision	recall	f1-score	support
dyed-lifted-polyps	0.92	0.84	0.88	93
dyed-resection-margins	0.83	0.87	0.85	55
esophagitis	0.82	0.84	0.83	101
normal-cecum	0.95	0.98	0.97	99
normal-pylorus	0.98	0.99	0.98	83
normal-z-line	0.80	0.78	0.79	85
polyps	0.94	0.97	0.96	100
ulcerative-colitis	0.93	0.89	0.91	45
accuracy			0.90	661
macro avg	0.90	0.89	0.89	661
weighted avg	0.90	0.90	0.90	661

Figure S6. Classification Report of MobileNetV2 for SAVE image dataset

S1.2 Mobile Net V3

MobileNetV3 further refines the V2 design with:

Neural Architecture Search (NAS): MobileNetV3 was designed using NAS to optimize for latency and accuracy trade-offs.

Squeeze-and-Excitation (SE) Modules: These allow the network to focus on the most informative features by learning channel-wise attention.

Hard-Swish Activation: A computationally efficient approximation of Swish activation that improves accuracy with negligible latency increase.

Two Variants:

MobileNetV3-Large: Optimized for maximum accuracy.

MobileNetV3-Small: Optimized for extreme efficiency in ultra-low-resource environments.

Because of their reduced computational cost and strong feature extraction capability, both MobileNetV2 and V3 are well-suited for deployment in embedded stereo vision systems, such as those used in construction site automation or real-time sand volume estimation.

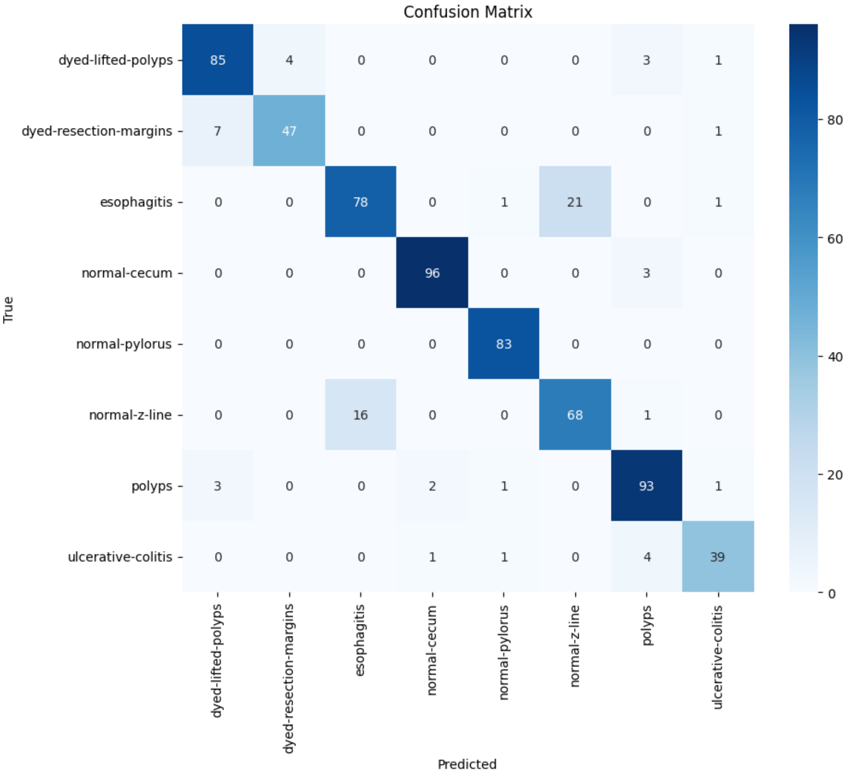

Figure S7. Confusion Matrix of MobileNetV3 for WLI image dataset

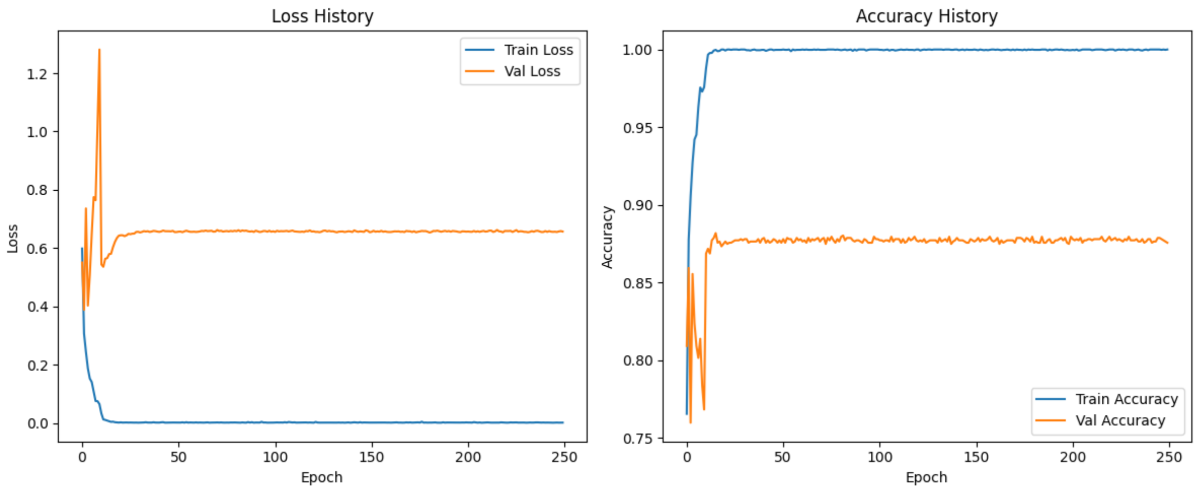

Figure S8. Loss and Accuracy History of MobileNetV3 for WLI image dataset

Classification Report:

	precision	recall	f1-score	support
dyed-lifted-polyps	0.89	0.91	0.90	93
dyed-resection-margins	0.92	0.85	0.89	55
esophagitis	0.83	0.77	0.80	101
normal-cecum	0.97	0.97	0.97	99
normal-pylorus	0.97	1.00	0.98	83
normal-z-line	0.76	0.80	0.78	85
polyps	0.89	0.93	0.91	100
ulcerative-colitis	0.91	0.87	0.89	45
accuracy			0.89	661
macro avg	0.89	0.89	0.89	661
weighted avg	0.89	0.89	0.89	661

Figure S9. Classification report of MobileNetV3 for WLI image dataset

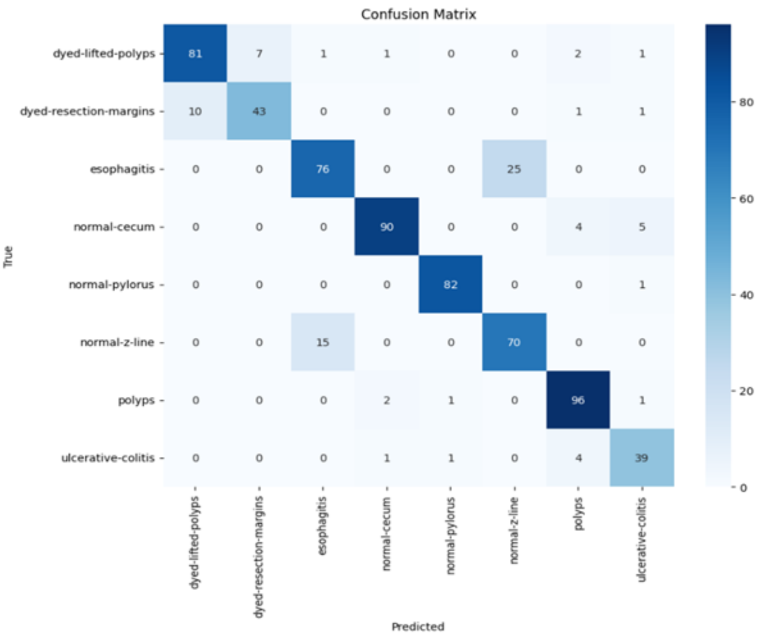

Figure S10. Confusion matrix of MobileNetV3 for SAVE image dataset

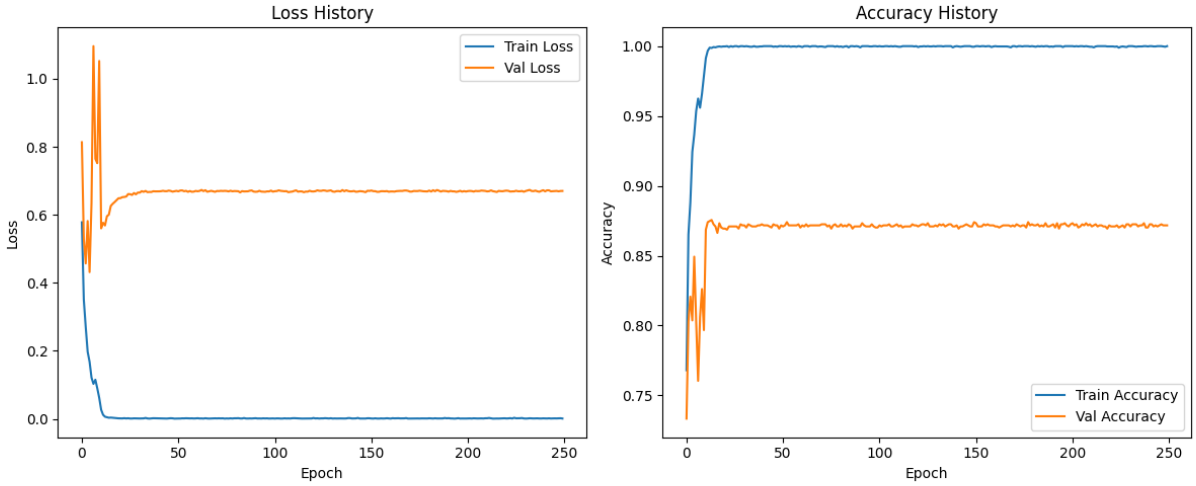

Figure S11. Loss and Accuracy of MobileNetV3 for SAVE image dataset

Classification Report:

	precision	recall	f1-score	support
dyed-lifted-polyps	0.89	0.87	0.88	93
dyed-resection-margins	0.86	0.78	0.82	55
esophagitis	0.83	0.75	0.79	101
normal-cecum	0.96	0.91	0.93	99
normal-pylorus	0.98	0.99	0.98	83
normal-z-line	0.74	0.82	0.78	85
polyps	0.90	0.96	0.93	100
ulcerative-colitis	0.81	0.87	0.84	45
accuracy			0.87	661
macro avg	0.87	0.87	0.87	661
weighted avg	0.87	0.87	0.87	661

Figure S12. Classification Report of MobileNetV3 for SAVE image dataset

S.1.3 ResNet50

ResNet-50 is a powerful deep convolutional neural network architecture that introduced the concept of residual learning, enabling the training of ultra-deep networks without suffering from the vanishing gradient problem.

Key Features:

Depth: ResNet-50 consists of 50 layers, structured as a stack of residual blocks, each containing convolutional layers and shortcut (identity) connections.

Residual Connections: These identity skip connections allow the network to learn the difference (residual) between the input and output of a block, improving convergence and accuracy.

Bottleneck Architecture: The 50-layer version uses a bottleneck design (1x1 → 3x3 → 1x1 convolutions) to reduce computational complexity while maintaining representational power.

Application Relevance:

Due to its strong feature representation and robustness, ResNet-50 is widely used in stereo vision pipelines for tasks like:

- Feature extraction in stereo matching
- Sand pile segmentation
- Object recognition and classification

While it offers higher accuracy than MobileNet variants, ResNet-50 demands significantly more computation and memory, making it more suited for desktop or GPU-based systems rather than edge devices.

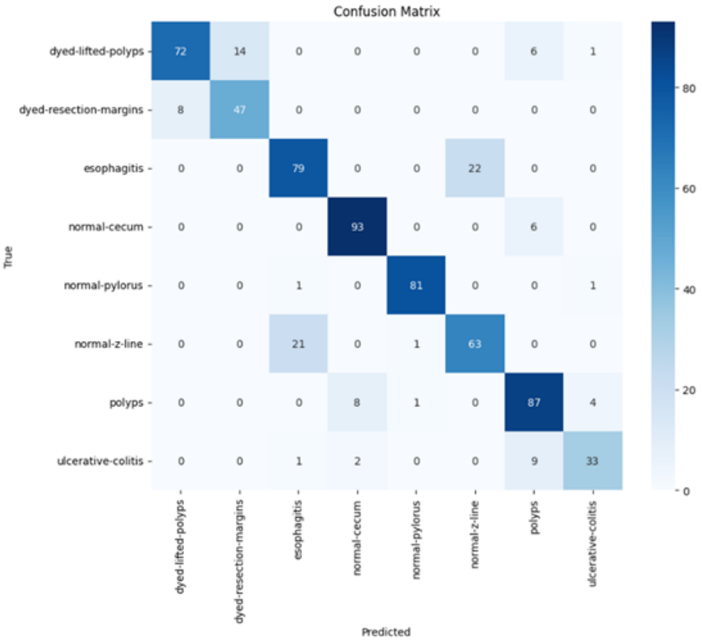

Figure S13. Confusion matrix of ResNet50 for WLI image dataset

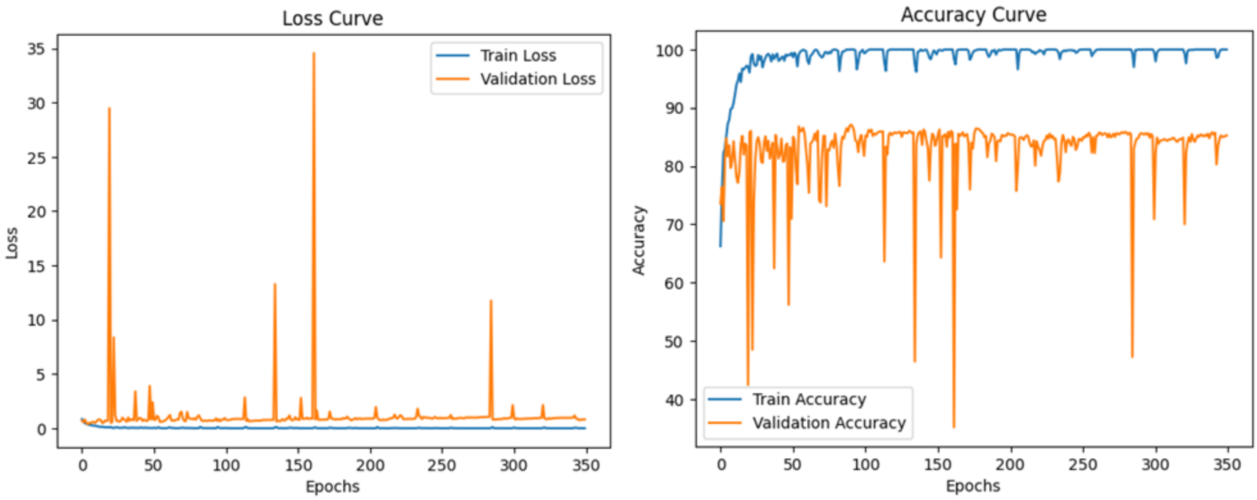

Figure S14. Loss and Accuracy of ResNet50 for WLI image dataset

Classification Report:

	precision	recall	f1-score	support
dyed-lifted-polyps	0.90	0.77	0.83	93
dyed-resection-margins	0.77	0.85	0.81	55
esophagitis	0.77	0.78	0.78	101
normal-cecum	0.90	0.94	0.92	99
normal-pylorus	0.98	0.98	0.98	83
normal-z-line	0.74	0.74	0.74	85
polyps	0.81	0.87	0.84	100
ulcerative-colitis	0.85	0.73	0.79	45
accuracy			0.84	661
macro avg	0.84	0.83	0.84	661
weighted avg	0.84	0.84	0.84	661

Figure S15. Classification Report of ResNet50 for WLI image dataset

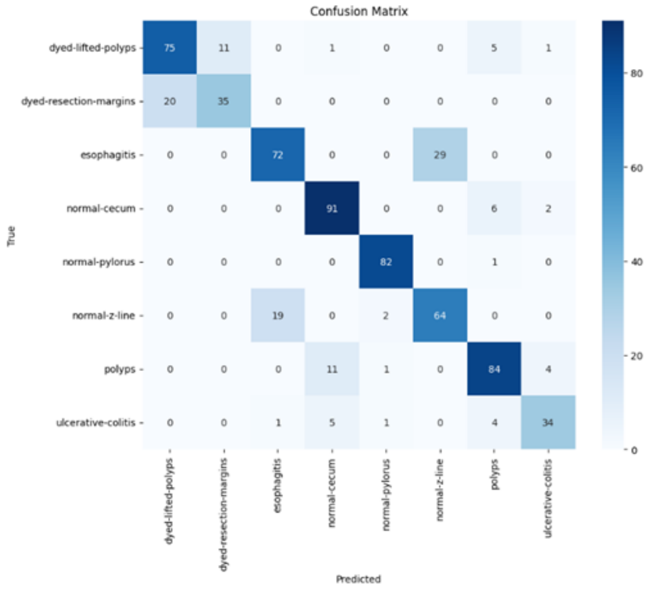

Figure S16. Confusion matrix of ResNet50 for SAVE image dataset

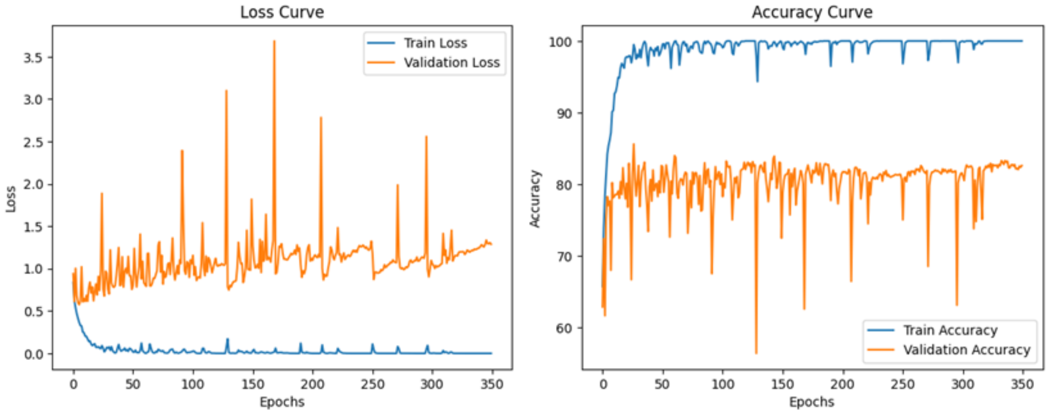

Figure S17. Loss and Accuracy History of ResNet50 for SAVE image dataset.

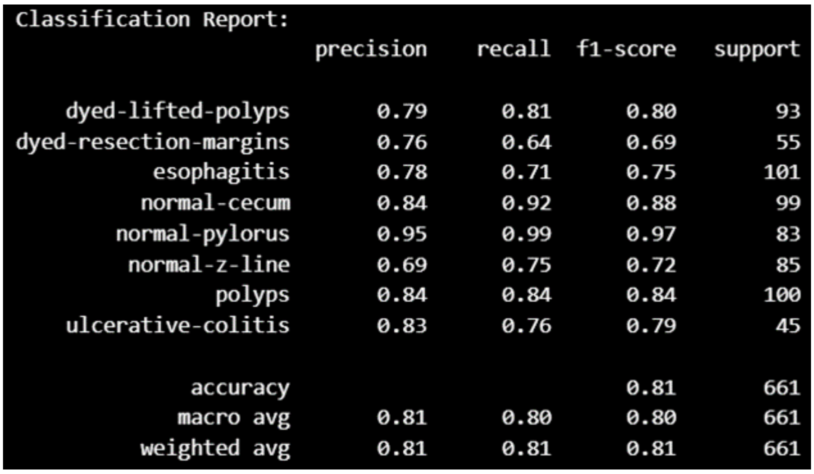

Classification Report:

	precision	recall	f1-score	support
dyed-lifted-polyps	0.79	0.81	0.80	93
dyed-resection-margins	0.76	0.64	0.69	55
esophagitis	0.78	0.71	0.75	101
normal-cecum	0.84	0.92	0.88	99
normal-pylorus	0.95	0.99	0.97	83
normal-z-line	0.69	0.75	0.72	85
polyps	0.84	0.84	0.84	100
ulcerative-colitis	0.83	0.76	0.79	45
accuracy			0.81	661
macro avg	0.81	0.80	0.80	661
weighted avg	0.81	0.81	0.81	661

Figure S18. Classification Report of ResNet50 for SAVE image dataset.

S1.4 AlexNet.

AlexNet, introduced in 2012, was the first deep convolutional neural network to demonstrate the significant potential of deep learning in large-scale image classification, notably winning the ImageNet Large Scale Visual Recognition Challenge (ILSVRC) with a substantial margin.

Key Features:

Architecture: Comprises 8 layers — 5 convolutional layers followed by 3 fully connected layers.

ReLU Activation: Replaced traditional sigmoid or tanh with the Rectified Linear Unit (ReLU), which accelerates convergence.

Overlapping Max Pooling: Used to reduce spatial dimensions while preserving spatial hierarchy and avoiding overfitting.

Dropout Regularization: Applied in the fully connected layers to mitigate overfitting.

GPU Utilization: Among the first models to be trained on GPUs, significantly reducing training time.

Application Relevance:

While AlexNet laid the groundwork for modern CNNs, its relatively shallow architecture and high parameter count make it less efficient for modern edge-based stereo vision tasks. However, it remains useful for:

Benchmarking newer models

Transfer learning in scenarios with limited data

Prototyping classical computer vision models in structured environments

Though surpassed in accuracy and efficiency by later architectures like ResNet and MobileNet, AlexNet's impact on CNN research and its simple structure still make it a foundational model in deep learning.

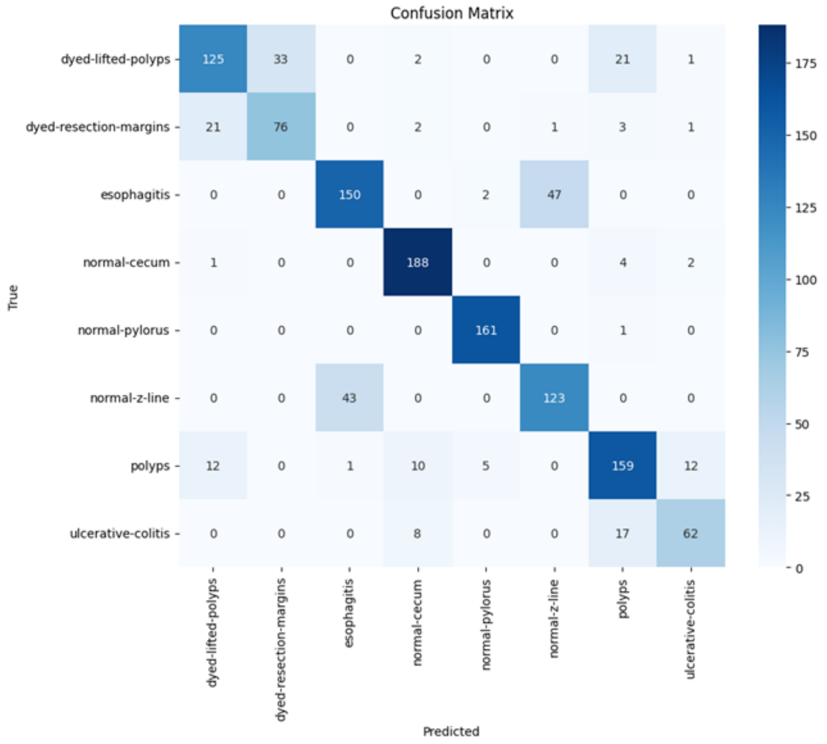

Figure S19. Confusion matrix of AlexNet's for WLI image dataset.

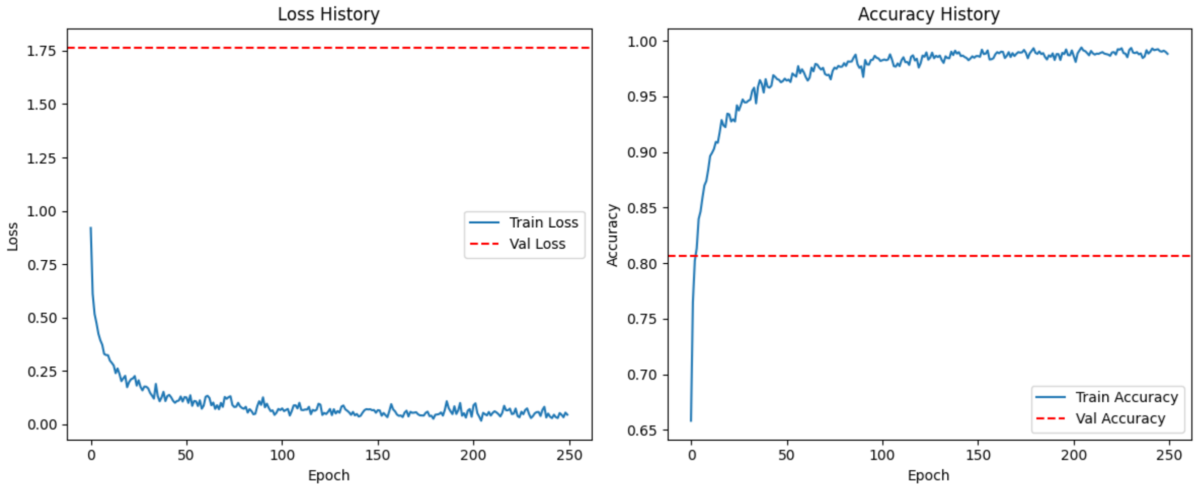

Figure S20. Loss and Accuracy History of AlexNet for WLI image dataset.

Classification Report:

	precision	recall	f1-score	support
dyed-lifted-polyps	0.79	0.69	0.73	182
dyed-resection-margins	0.70	0.73	0.71	104
esophagitis	0.77	0.75	0.76	199
normal-cecum	0.90	0.96	0.93	195
normal-pylorus	0.96	0.99	0.98	162
normal-z-line	0.72	0.74	0.73	166
polyps	0.78	0.80	0.79	199
ulcerative-colitis	0.79	0.71	0.75	87
accuracy			0.81	1294
macro avg	0.80	0.80	0.80	1294
weighted avg	0.81	0.81	0.81	1294

Figure S21. Classification report of AlexNet for WLI image dataset.

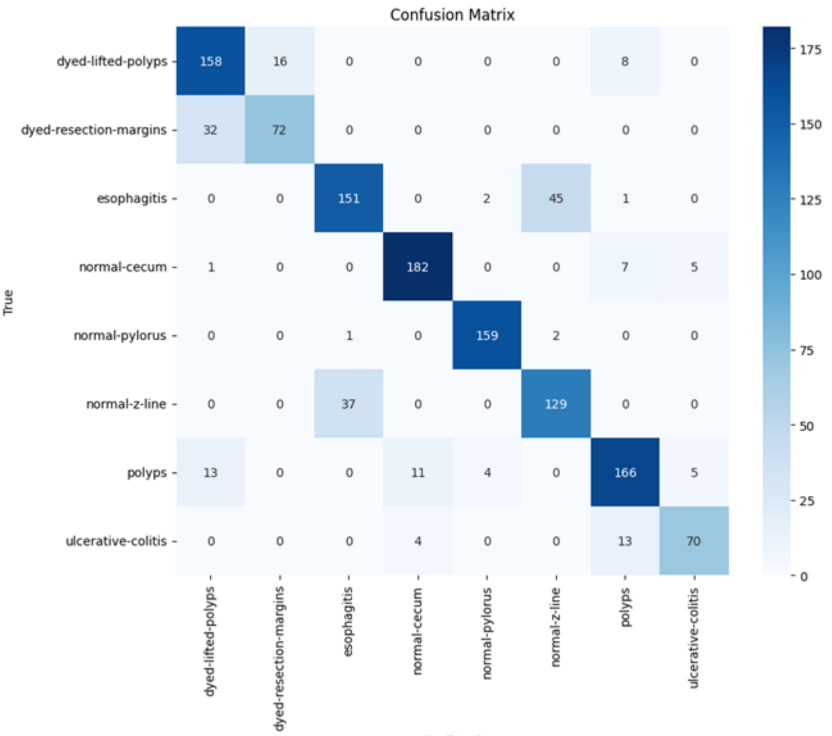

Figure S22. Confusion matrix of AlexNet for SAVE image dataset

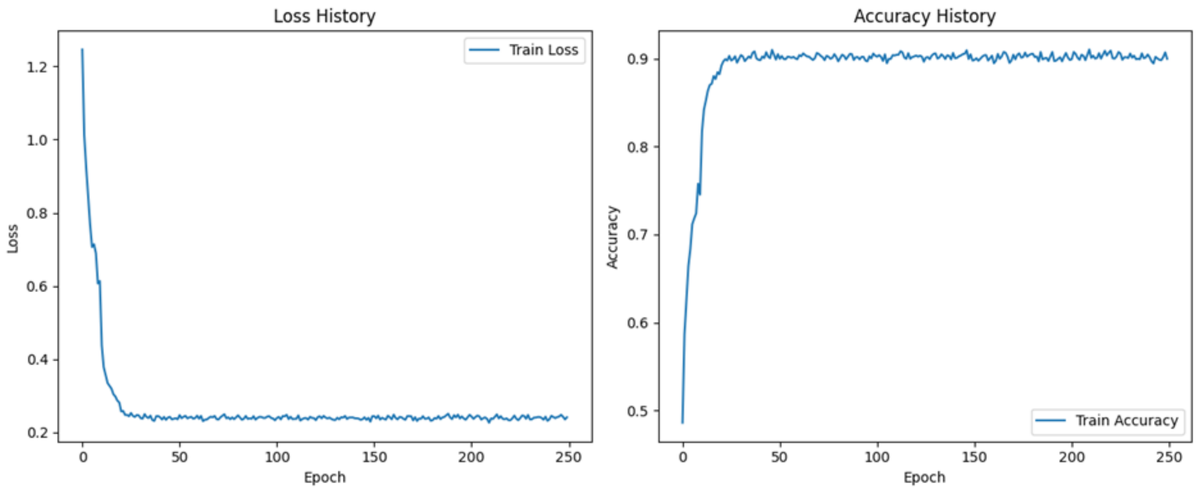

Figure S23. Loss and Accuracy of AlexNet for SAVE image dataset.

Classification Report:

	precision	recall	f1-score	support
dyed-lifted-polyps	0.77	0.87	0.82	182
dyed-resection-margins	0.82	0.69	0.75	104
esophagitis	0.80	0.76	0.78	199
normal-cecum	0.92	0.93	0.93	195
normal-pylorus	0.96	0.98	0.97	162
normal-z-line	0.73	0.78	0.75	166
polyps	0.85	0.83	0.84	199
ulcerative-colitis	0.88	0.80	0.84	87
accuracy			0.84	1294
macro avg	0.84	0.83	0.84	1294
weighted avg	0.84	0.84	0.84	1294

Figure S24. Classification report of AlexNet for SAVE image dataset

S2. SAVE Results

S2.1 Detailed Definitions and Units

• C (Calibration Coefficient Matrix)
- What it is: The 3×N matrix mapping N-band spectral measurements to CIE 1931 XYZ tristimulus space.
- Dimensions: 3×N.
- Units: Dimensionless gains (scaling each spectral band to reproduce XYZ coordinates).

• XYZ_spectrum (Reference Tristimulus Values)
- What it is: A 3×M matrix of CIE 1931 tristimulus values [X,Y,Z] for M Macbeth Color Checker patches under the target illuminant.
- Dimensions: 3×M.
- Units: Unitless (normalized to 0–1).

• V (Measured Spectral Responses)
- What it is: An M×N matrix of measured spectral responses; each row corresponds to one Macbeth patch, each column to one sampled wavelength/band.
- Dimensions: M×N.
- Units: Spectral radiance or irradiance.

• Square Brackets "[ ]"
 – Denote matrix assembly or grouping; indicates the matrix of those values.
 – Not a function, just notation for the matrix itself.

• pinv(V)
 – The Moore–Penrose pseudo-inverse of V.
 – Provides the least-squares solution to V x = b for non-square or rank-deficient matrices.
 – Computed via $(V^T V)^{-1} V^T$ (if full column rank) or via SVD to satisfy pseudo-inverse conditions.

• XYZ_correct (Reconstructed Tristimulus Matrix)
– What it is: The predicted CIE 1931 tristimulus values obtained by applying the calibration coefficient matrix C to the measured spectral response matrix V.
– Dimensions:
- If V is M × N (M samples, N spectral bands), then [XYZ_correct] is 3 × M (each column is $[X\_i, Y\_i, Z\_i]^T$ for sample i).
- For a single measurement vector v (N × 1), XYZ_correct is a 3 × 1 column vector.
– Units: Same as the reference XYZ values in [XYZ_spectrum], unitless normalized values (range 0–1) representing CIE tristimulus coordinates under the chosen illuminant.

• S(λ): Spectral power distribution of the illuminant
– Units: normalized unitless.
– Role: Describes light source intensity at each wavelength λ.
• R(λ): Spectral reflectance (or transmittance)
– Units: Unitless fraction (0–1).
– Role: Fraction of incident light at λ reflected by the object.
• $\bar{x}(\lambda)$, $\bar{y}(\lambda)$, $\bar{z}(\lambda)$: CIE 1931 2° Standard Observer color matching functions
– Units: Unitless sensitivity functions.
– Role: Weight the SPD×reflectance to mimic human eye response for X, Y (luminance), Z channels.
• k: Normalization constant
– Defined as $k = 100 / \int_{400}^{700} S(\lambda)\, \bar{y}(\lambda)\, d\lambda$
– Ensures Y = 100 for a perfect diffuser (R(λ)=1 ∀ λ).

• V_color
– Description: The sensor's linear color response matrix. Represents the raw spectral signal measured when illuminated by the 24-color patch board. Each entry corresponds to the linear (proportional) output of each spectral band for each patch.
– Role: Captures the baseline, color-dependent response before any non-linearities or dark current are considered.
• V_non-linear
– Description: The non-linear response component of the sensor. Accounts for known sensor behaviors such as gamma correction, sensor saturation, or other non-linear transfer functions.
– Role: Models deviations from linearity, ensuring that the final standardized matrix corrects for both linear and non-linear characteristics.
• V_dark
– Description: The dark current or baseline offset component. Measured by capturing a "dark frame" (no illumination) and representing the sensor's zero-light output (noise floor).
– Role: Subtracted or standardized alongside V_color and V_non-linear to remove baseline noise and ensure accurate colorimetric conversion.

M (Transformation Matrix)
• Definition: The optimized transformation matrix that maps the sensor's raw color-channel responses to the desired calibrated output scores.
• Dimensions: If [Score] is (P × N) and [V_Color] is (N × B), then [M] is (P × B), where:
– P = Number of output channels (3 for X, Y, Z).
– B = Number of sensor color bands (24 patches or spectral bands).
• Role: Enables direct computation of calibrated color scores from new raw measurements

Score (Target Output Matrix)

• Definition: The target output matrix containing the reference colorimetric scores for each calibration sample. Typically these are the corrected tristimulus values (XYZ) measured by a reference spectrometer or obtained after initial regression steps.

• Dimensions: P × N, where:

– P = Number of output channels (3 for X, Y, Z).

– N = Number of calibration samples (number of color patches = 24).

• Units: The same units as the color scores, for example unitless normalized XYZ values (0–1).

• x (Corrected/Normalized Value)

– What it is: The output variable after applying the non-linear adjustment (e.g., corrected reflectance or normalized sensor response).

– Units: Dimensionless (normalized scale, typically 0–1).

• x_0 (Midpoint/Threshold Value)

– What it is: The center or inflection point of the logistic (sigmoid) function, representing the input value at which the output reaches 50% of its dynamic range.

– Units: Same as x (dimensionless normalized input scale).

• γ (Gamma, Steepness Parameter)

– What it is: Controls the slope or steepness of the sigmoid curve around x_0. A larger γ yields a sharper transition; a smaller γ produces a gentler curve.

– Units: Reciprocal of x. It quantifies the rate of change per unit of input.

The data generated through simulation were utilized for the initial evaluation of the system's performance. The characterization involved measuring their spectrum emissions, while also taking into account the sensitivities provided by the camera manufacturers. The spectral curves of the 24-color Macbeth Color Checker chart were utilized for both the training and validation sets of the samples. Table S1 displays the RMSE measurements for each of the 24 hues.

Table S1. RMSEs of the XYZ values before and after calibration.

S.no	Before calibration			After Calibration			RMSE	SD
	X	Y	Z	X	Y	Z		
1	10.96	9.92	4.63	11.14	9.87	4.26	0.24	0.30
2	38.74	35.80	18.65	38.57	35.94	18.66	0.13	0.08
3	16.62	19.07	24.13	16.48	18.79	24.11	0.18	0.17
4	10.33	12.86	4.62	10.16	13.03	4.85	0.19	0.19
5	24.05	23.87	31.55	24.16	24.07	31.60	0.13	0.08
6	30.12	42.15	32.40	30.10	42.17	32.42	0.02	0.002
7	38.10	30.24	4.28	38.04	30.37	4.22	0.09	0.04
8	11.70	11.47	25.90	11.64	11.37	25.91	0.07	0.02
9	29.01	19.91	9.62	29.20	19.78	9.60	0.13	0.08
10	8.26	6.49	9.63	8.06	6.49	9.86	0.18	0.17
11	34.15	44.06	8.44	34.15	44.02	8.53	0.06	0.01
12	47.99	44.55	6.05	48.05	44.34	6.17	0.15	0.11

13	6.82	5.79	21.07	6.90	5.91	21.00	0.09	0.04
14	14.55	23.55	7.22	14.58	23.51	7.12	0.07	0.02
15	21.08	12.25	3.57	21.01	12.28	3.65	0.06	0.01
16	58.40	60.69	7.54	58.38	60.79	7.42	0.09	0.04
17	28.98	19.54	20.67	28.94	19.52	20.66	0.02	0.002
18	12.81	19.01	28.54	12.84	19.10	28.56	0.05	0.01
19	82.12	88.54	67.20	82.31	88.73	67.51	0.24	0.30
20	54.74	58.92	45.52	54.28	58.40	44.75	0.60	1.89
21	33.08	35.73	27.24	33.26	35.82	27.54	0.21	0.23
22	18.18	19.62	14.94	18.86	20.31	15.62	0.68	2.43
23	9.13	10.01	8.13	8.56	9.26	7.21	0.76	3.04
24	2.87	3.19	2.39	3.10	3.35	2.68	0.23	0.27
Average							0.19	0.39

The calibration of the camera is a crucial component of the SAVE algorithm. Figure S25 displays the color disparity outcomes prior to and following calibration. Following the calibration of the camera, the color exhibited a striking resemblance to the color acquired by the spectrum analyzer, rendering the distinction challenging to perceive. Prior to camera calibration, the mean chromatic aberration of all 24 color blocks was 10.76. After calibrating the camera, the average chromatic aberration decreased to a minimum of 0.63.

S.no	Before Camera Calibration	Spectrometer	Chromatic Aberration	After Camera Calibration	Spectrometer	Chromatic Aberration
1			7.08			1.24
2			7.63			0.78
3			16.43			0.86
4			12.45			1.68
5			14.92			0.45
6			10.80			0.05
7			7.47			0.52
8			18.46			0.22
9			13.19			0.62
10			8.09			1.30
11			8.03			0.09
12			6.43			0.58
13			10.32			0.30
14			12.19			0.23
15			13.31			0.17
16			7.00			0.18
17			17.80			0.03
18			22.22			0.19
19			0.00			0.08
20			5.30			0.30
21			9.77			0.42
22			12.71			0.81
23			13.34			2.01
24			3.37			1.96
	Average Color Difference		10.76	Average Color Difference		0.63

Figure S25. The color difference before and after camera calibration

In Figure S25, the reflectance values of the six primary colors inside the 24-color block are illustrated. These colors are blue (13), red (15), green (14), yellow (16), magenta (17), and cyan (18). Based on the analysis of the 24-color blocks, it was noted that the red block exhibited the most significant disparity between the simulated and actual reflectance values, particularly across the longer wavelength range of 600 to 780 nm. One of the limitations of the study is considered to be this element. All of the remaining 23 color blocks exhibited RMSEs below 0.1, with the color black demonstrating the lowest RMSE of 0.015. The RMSE was merely 0.056, suggesting that the majority of the color could be replicated with precision.

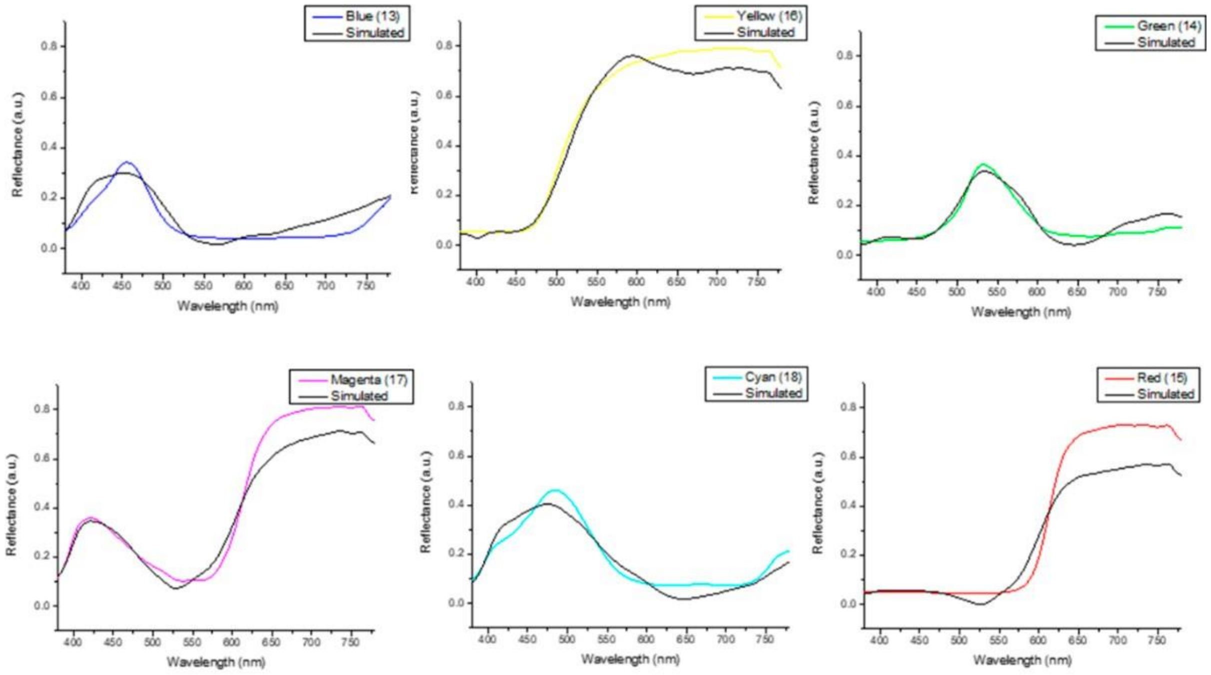

Figure S26. RMSEs between analog and measured spectra of each color block

RMSE values can be graphically and mathematically represented by calculating the disparity between simulated and measured colors. The representation of a color can be denoted as LAB, where L represents lightness, channel A, and channel B, respectively. The numerical definition of any color can be achieved by manipulating the values of L, A, and B. The L, A, and B values of the simulated and computed colors are depicted in Figure S27. The average color disparity was about 0.75, suggesting that the replicated color was visually precise.

Measured Color				Simulated Color				Color Difference
L	a	b	Color	L	a	b	Color	
37.61	13.65	24.56		37.64	11.87	22.60		1.32
66.48	14.68	31.10		66.37	15.47	30.58		0.73
50.44	-7.58	-6.44		50.72	-8.16	-6.09		0.75
42.80	-16.14	30.50		42.48	-14.04	31.19		1.50
56.16	5.70	-8.01		55.96	5.89	-8.51		0.43
70.99	-34.14	16.44		71.01	-34.00	15.65		0.41
61.97	32.36	66.76		61.85	33.07	65.32		0.80
40.20	6.07	-27.03		40.27	6.15	-26.67		0.27
51.59	46.04	27.52		51.72	44.60	27.21		0.52
30.62	18.70	-9.45		30.52	20.85	-8.70		1.46
72.24	-24.91	66.55		72.23	-25.21	66.79		0.13
72.46	17.04	75.67		72.59	16.33	76.17		0.49
29.18	13.90	-37.66		28.70	15.23	-38.14		0.81
55.59	-40.93	42.88		55.56	-41.82	42.54		0.42
41.66	53.78	34.95		41.60	54.34	34.25		0.51
82.26	1.48	87.73		82.21	1.96	87.67		0.27
51.29	46.36	1.08		51.30	46.12	0.95		0.10
50.80	-31.41	-12.85		50.66	-30.83	-13.20		0.42
95.47	-3.88	21.64		95.38	-3.70	22.54		0.52
80.96	-3.08	18.47		81.32	-3.39	17.74		0.63
66.38	-2.74	15.56		66.33	-3.33	15.44		0.69
52.18	-2.26	12.86		51.36	-2.59	12.85		0.91
36.47	-2.05	9.55		37.78	-3.18	8.64		2.00
21.40	-1.45	6.28		20.70	-2.87	7.50		2.02
							Average Color Difference	0. 75

Figure S27. LAB values of the simulated and observed colors.
